# Real-World Evidence to Support the Registration of a New Osteoporosis Medicinal Product in Europe

**DOI:** 10.1007/s43441-024-00616-7

**Published:** 2024-02-10

**Authors:** Colleen Davenport, Patricia Gravel, Yamei Wang, Setareh A. Williams, Alethea Wieland, Bruce Mitlak

**Affiliations:** 1 Radius Health, Inc., 22 Boston Wharf Road, 7th Floor, Boston, MA 02210 USA; 2 Triskel Integrated Services, Le Grand-Saconnex, Switzerland; 3 Star Biopharma Consulting, Malvern, PA USA; 4 Clinical Research Strategies, Wexford, PA USA

**Keywords:** Tymlos, Eladynos, Abaloparatide, Osteoporosis, Real-world evidence, Real-world data, Regulatory decision making, EMA, FDA

## Abstract

Real-World Evidence (RWE), which has historically been used to support post-approval safety studies, has recently gained acceptance for new drug applications as supportive evidence or as new clinical evidence for medicinal products with orphan designation and/or in disease areas with high unmet need. Here, we present a case study for the use of RWE in the approval of abaloparatide in the European Union (EU) under the tradename Eladynos. In addition to data from the pivotal Phase 3 study, the marketing authorization application (MAA) included clinical data from additional interventional and observational studies, as well as post-marketing data obtained from the United States (US) market since approval of abaloparatide by the Food and Drug Administration (FDA) in 2017. The new interventional studies were not designed to assess fracture efficacy and cardiovascular safety which were topics of concern raised by the Committee for Medicinal Products for Human Use (CHMP) during their review of the initial MAA submitted in 2015. However, these studies taken together with the RWE formed the basis for a new MAA. Prior to the planned resubmission in the EU, national Scientific Advice (SA) was sought on the proposed clinical program, specifically on the relevance of Real-World Data (RWD) derived from an observational study to support and complement the efficacy and safety data already available from prospective randomized clinical trials. This case study demonstrates successful use of RWE to address a previously identified gap raised by the CHMP during the review of an earlier MAA, which led to the approval of Eladynos for the treatment of osteoporosis in the EU.

## Introduction

Randomized controlled trials (RCT) are the gold standard for the assessment of clinical efficacy and safety in regulatory decisions. However, the ability to fully characterize the safety of a new medicinal product in the limited setting of a controlled trial has led to the collection of patient data from post-approval safety surveillance programs for decades. Over time, the value of this “real-world” data (RWD) beyond pharmacovigilance studies has increasingly been recognized and includes data derived from electronic health records, disease registries, and patient surveys. Clinical evidence about the benefits and risks of a medicinal product derived from RWD is referred to as Real-World Evidence (RWE). More recently, regulators have considered RWE in the context of their benefit–risk evaluation of new products and/or indications, but mostly for drugs with orphan drug designations and/or in disease areas with high unmet medical need [1]. In the first part of this article, an overview of the current regulatory RWE framework and existing guidance in the EU is presented followed by a case study for which RWE was used to address concerns regarding the safety and efficacy of abaloparatide for the treatment of osteoporosis in postmenopausal women at increased risk of fracture following a previously rejected application.

## Use of Real-World Data/Real-World Evidence in EU Medicines Regulation

The European Medicines Agency (EMA) works together with the Heads of Medicines Agencies (HMA) to form a European medicines regulatory network that focuses on the development, co-ordination, and consistency of the European medicines system while addressing key strategic initiatives for the network. To this end, the EMA and HMA have issued strategic 5-year roadmaps/network strategies that identify shared challenges, goals, and priorities, to ensure the continued success of the European medicines regulatory network since 2005. A key goal of each of these 5-year roadmaps is to further strengthen the protection of human health while encouraging and facilitating innovation and research in the EU (see Table 1). The first Roadmap focused on strengthening and operationalizing the partnership of all the EU regulatory authorities, introduction of new legal tools to accelerate patient access to medicine and creation of a robust pharmacovigilance system across the EU (see Table 1). The concept of real-world use of medicines first appeared in the “Roadmap to 2015” published in 2010. A key strategic priority included in this roadmap focused on minimizing the risks to public health when using newly approved medicines in a “real-world” setting by building on the pharmacovigilance platforms introduced in the “Roadmap to 2010” (see Table 1). Real-world data from registries, claims, and electronic health records were some of the initial resources used in post-marketing pharmacovigilance activities such as signal detection, validation, and assessment.

**Table 1.** Roadmap to Consideration of Real-World Evidence in Regulatory Applications.

Year Published	EMA Roadmap	Strategic Priorities	Current Regulatory Environment/Challenges Driving Priorities	Key Accomplishments
2005 [3]	Roadmap to 2010	Contributing to better protection and promotion of public animal health Improving the regulatory environment for medicinal products Helping to stimulate innovation, research, and development in the EU	Enlargement of EU on 1 May 2004 adding 10 new countries (Czech Republic, Estonia, Cyprus, Latvia, Lithuania, Hungary, Malta, Poland, Slovakia, and Slovania) to the original 6 (Belgium, France, Germany, Italy, Luxembourg, and Netherlands) Entry into force new Community pharmaceutical legislation by November 2005 [Directive 2004/27/EC established the Co-ordination group for Mutual recognition and Decentralized procedures—human (CMDh)]	Strengthened the partnership between all EU regulatory authorities through establishment of a network of excellence at the EU level Close cooperation between EMA and HMA via joint initiatives Introduction of Risk Management Plans Strengthened drug monitoring system through enhancement of EudraVigilance system Introduction of new legal tools including conditional marketing authorization, accelerated assessment, and compassionate use Implementation of new pediatric legislation
2010 [4]	Roadmap to 2015	Addressing public health needs Accelerating the development of medicines for unmet need and rare diseases Facilitating access to medicines Encourage use of Scientific Advice (SA) and promote early interaction between regulators and sponsors Optimizing the safe and rational use of medicines Minimizing the risks to public health that are inherent in the “**real-world” use of medicines** Improved risk-minimization tools	Growing importance of patients’ and health care professionals’ voice in benefit/risk considerations Increasing scope of Agency’s role and responsibilities to generics, non-prescription medicines, medical devices, falsified medicines, and pharmacovigilance Demographic challenges including aging population Public health threats including antimicrobial resistance and emergence of new diseases Rapid development of new technology and emerging science such as personalized medicine, advanced therapies, and nanotechnology Continued increase in the importance of globalization over time Demands for greater transparency and openness	EU Clinical Trials Register launched in March 2011; sponsors required to post clinical trial results in July 2014 Legislation on falsified medicines adopted in July 2011 and entered into force in January 2013 New pharmacovigilance legislation became operational in July 2012 Launch of European database of suspected adverse drug reactions in 2012 (www.adrreports.eu) Creation of Pharmacovigilance Risk Assessment Committee (PRAC) Establishment of the Article 57 database—first EU database of all authorized medicines Adoption of EMA policy on publication of clinical data in October 2014 which set a new standard for transparency in public health and pharmaceutical research and development
2015 [5]	Roadmap to 2020	Contributing to human health with an emphasis on availability of medicines, timely access to new medicines, support for patient-focused innovation and increased transparency Optimizing the operation of the network through reinforcement of the scientific and regulatory capacity of the network, ensuring effective communication and increased collaboration with other authorities and stakeholders Contributing to the global regulatory environment through assurance of product supply and data integrity, convergence of global standards, promotion of mutual reliance, and work-sharing and training	Brexit Greater involvement of patients in the work of regulatory authorities Further globalization necessitating collaboration with an even broader network of regulators Societal trends including aging population, population migration, polypharmacy, and comorbidity Rapidly increasing costs and complexity of developing new medicines leading to society asking for more in terms of timely access to novel treatments Increasing emphasis on benefit-risk balance throughout the medicine’s life cycle Need to improve access to medicines by ensuring a robust supply chain and supporting development of generics and biosimilars	Launch of PRIME (priority medicines) in March 2016 Introduction of parallel SA with HTA bodies Workshop on the opportunities and challenges associated with the use of big data in medicine development and regulation organized by the EMA in November 2016. **HMA/EMA Big Data Task Force publishes report on regulatory landscape of big data and opportunities for improvement** Launch of Patient Registry initiative to support a more systematic approach to the contribution of existing disease registries to the benefit-risk evaluation of medicines Establishment of Regulatory Science Observatory (RSO) to identify and meet emerging trends in science and technology and promote the strategic application of science in the regulation of pharmaceutical products New Medical Device Regulation—EU 2017/745 for medical devices
2020 [6]	Roadmap to 2025	Availability and accessibility of medicines Data analytics, digital tools, and digital transformation Innovation Antimicrobial resistance and other emerging health threats Supply-chain challenges Sustainability of the network and operational excellence	COVID-19 Dramatic increase in the pace of innovation and volume of complex medicines Advances in genomic sequencing techniques leading to a move away from broad, one-size-fits-all indications to narrower and more precise medicines (personalized medicine) **Increasing focus on “Big Data” including data from Real-World Populations necessitating regulators to address challenges arising from collecting and processing data from patients** “Quantified Self” movement becoming more integrated into clinical development using wearable digital devices Cyber Security	Coordinated response to COVID-19 pandemic Launch of new Clinical Trial Information System enabling the centralized application and management of clinical trial data in the EU New Clinical Trials Regulation (CTR) comes into force Accelerating Clinical Trials in the EU (ACT EU) initiative launched by EMA and HMA **DARWIN EU® Advisory Board formed in June 2021 and contract awarded to Erasmus University Medical Centre Rotterdam in February 2022** Contribution to ongoing ICH discussion on guidelines for Patient Experience Data in medicines regulation including the ICH Reflection Paper on Patient-Focused Drug Development (PFDD) New Medical Device Regulations come into force May 2021

Early in 2019, the HMA-EMA Joint Big Data task force published recommendations supporting acceptability of evidence derived from “Big Data” [2] (see Table 1). Big data consist of large and often complex datasets that tend to be both unstructured and heterogeneous. Big data accumulate rapidly and need to be analyzed computationally to reveal patterns, trends, and associations. The currently established regulatory framework is based on the assessment of data from well-controlled, randomized clinical trials designed to provide unbiased estimates of efficacy and safety. Therefore, the introduction of big data required thoughtful assessment of when and how such data may be considered for regulatory decision making in the product life cycle. The initial priority of the task force was to develop global standards for data quality and methodology for the use of big data in regulatory decision making.

By 2020, big data had become part of the current regulatory environment necessitating regulators to address challenges arising from collecting and processing such data (see Table 1). The EMA’s Data Analysis and Real-World Interrogation Network (DARWIN EU) was created to provide timely and reliable evidence on safety and effectiveness of medicines for human use using real-world healthcare databases across the EU. The objective of DARWIN EU is to facilitate the exchange of healthcare data for use in research, regulatory policy making, and healthcare delivery in Europe. The DARWIN EU® Advisory Board, formed in June 2021, brings together a federated network of data partners from public or private, regulators, patients, health technology assessment (HTA) bodies, and payers. Erasmus University Medical Centre based in Rotterdam, Netherlands was selected in February 2022 as the service provider to deliver DARWIN EU.

The network completed its first four studies in 2022, and the EMA is planning between 10 and 15 studies in 2023, and around 150 per year from 2025 [7]. The protocols and results of these studies are publicly available in the EU PAS (Post-Authorisation Studies) Register (https://www.encepp.eu/encepp/studiesDatabase.jsp). Once fully operational, DARWIN will answer questions posed by EMA’s scientific committees and National Competent Authorities to better understand diseases, populations and the use, safety, and effectiveness of medicines. The EMA recently issued a report on the experience gained from regulator-lead real-world studies from September 2021 to February 2023 [8]. To determine the impact of these studies on regulatory decision making, the EMA conducted a survey of the regulators requesting the studies and of the 18 responses, 12 of these studies were considered supportive [PRAC review of safety signals and PSURs (*n* = 7), scientific advice requests (*n* = 3), and PDCO decisions on PIP/waiver requests (*n* = 2)].

Currently the EMA is working to develop a data quality framework for all data used in regulatory decision making including RWD [1]. The EMA is also evaluating the evidentiary value of RWE and emphasizes a complementary evidence approach with a place for both RCT and RWE to be used in conjunction rather than in opposition. EMA’s vision is that by 2025, the use of RWE across a spectrum of regulatory use cases will have been realized. While broad use of RWE in regulatory decision making is not yet a reality, use of RWE to support safety of drugs and more recently initial marketing authorizations have been observed. Flynn et al. reported for new MAA submitted in the EU (centralized procedure) between 1 January 2018 and 31 December 2019, 63 of 158 (39.9%) of initial applications contained RWE and approximately one third of these applications included RWE collected prior to the planned authorization [9]. These RWDs were primarily from registry (60.3%) and hospitals data (31.7%) and were mainly included to support safety (87.3%) and efficacy (49.2%). The most common study design was a cohort study (87.6%). Bakker et al. further characterized this dataset but focused on MAAs in which the RWE contributed to the efficacy/effectiveness data [10]. Of the 63 MAAs with RWE, 32 (50%) included RWD related to efficacy/effectiveness outcomes and disease epidemiology. Two thirds of those applications were for products with orphan designation and were primarily for indications with high unmet medical need. From the compilation of MAAs submitted between 2018 and 2019, only 3% (5/158) contained RWE collected prior to the authorization and for which the submitted efficacy data were considered by the CHMP as supportive for their regulatory decision making. These 5 medicinal products included onasemnogene abeparvovec (Zolgensma), trientine dihydrochloride (Cufence), melphalan (Phelinun), and hydroxycarbamide (Xromi). The fifth product was for a rare thromboembolic disorder and was withdrawn. A few details from the European Public Assessment Reports (EPAR) on how the CHMP relied on the RWE for regulatory decision making for these products are provided below.

Two of these products (melphalan and hydroxycarbamide) were submitted as hybrid applications and relied on nonclinical and clinical data from a reference product already approved in the EU. Mephalan is a cytotoxic agent that works by preventing cell division. To support the new indication, use as a conditioning treatment prior to allogeneic hemopoietic stem cell transplantion, the sponsor reviewed safety and efficacy data from 18 peer-reviewed articles published between 2005 and 2018 including 3096 patients from both interventional and observational studies. Similarly, hydroxycarbamide had a well-established safety and efficacy profile as it had been used in the EU for many years. A literature review containing real-world studies with effectiveness and safety data was considered adequate to support the indication for the prevention of vaso-occlusive complications of sickle cell disease. The other two products (onasemnogene abeparvovec and trientine dihydrochloride) were for orphan diseases. Onasemnogene abeparvovec initially received conditional approval for treating spinal muscular atrophy. The pivotal Phase 3 study included a single arm and relied on a natural real-world historical cohort control (external comparator) for demonstration of efficacy. Of note, the use of a historical comparator from the natural history study was agreed upon during a scientific advice meeting. During the MAA review, the CHMP considered the historical comparator adequate owing to the similar timing of outcome/endpoint assessment in the Phase 3 study compared to the natural real-world history cohort, and to the homogeneity of the cohorts. The safety was augmented by inclusion of post-marketing experience from the US and France including 192 cases. A Post-Approval Efficacy Study was required as a condition for approval. Trientine dihydrochloride was submitted as a full mixed marketing application. The primary safety and efficacy data were derived from a Phase 4 real-world study that reviewed the medical records from 77 patients. In addition, trientine had been used for over 30 years to treat Wilson Disease and a review of literature provided additional supportive information. During the MAA review, several methodological shortcomings were identified by the CHMP (e.g., lack of controls, blinding/randomization, lack of sample size calculation, lack of definition of the primary endpoint), but these did not prevent the approval of the product considering the totality of the evidence provided on the efficacy and safety.

A recent paper outlines the benefits and limitations of RWE studies and how to ensure that transparent and high-quality evidence is generated, with a focus on osteoporosis research [11].

A Case Study is presented in the following section for which regulators considered RWE in the context of their benefit–risk evaluation of a new medicinal product for osteoporosis.

## Case Study

### Osteoporosis and Available Therapy

Osteoporosis is a systemic skeletal disease characterized by low bone mass and microarchitectural deterioration of bone tissue, with a consequent increase in bone fragility and susceptibility to fracture [12–14]. The main characteristics of osteoporosis are low bone mineral density (BMD) and fractures. Two classes of therapies for treatment of osteoporosis are currently available in the EU, the antiresorptives (bisphosphonates and denosumab) and those with anabolic activity (teriparatide, abaloparatide, and romosozumab). Antiresorptives increase BMD by reducing the ability of osteoclasts to resorb bone, while the anabolic drugs act on osteoblasts to build new bone.

Abaloparatide is a synthetic 34 amino acid peptide that shares 41% homology to parathyroid hormone [PTH(1–34)] and 76% homology to parathyroid hormone related peptide [PTHrP(1–34)] and is a potent and conformation specific activator of the PTH1 receptor signaling pathway. Once daily administration of abaloparatide stimulates new bone formation on trabecular and cortical (periosteal, intercortical, and endocortical) bone surfaces by preferential stimulation of osteoblastic activity over osteoclastic activity. Teriparatide (trade name Forsteo in the EU and Forteo in the US), a recombinant human parathyroid hormone peptide (rhPTH1-34), is the closest comparator to abaloparatide and was used as the active comparator (open-label) in the pivotal registration placebo-controlled Phase 3 study (BA058-05-003; ACTIVE) [15].

The development of abaloparatide for the treatment of osteoporosis in postmenopausal women followed the recommendations set forth in the Guideline on the Evaluation of Medicinal Products in the Treatment of Primary Osteoporosis [16]. Consistent with the Guideline, SA provided before the initial MAA emphasized the need to show efficacy on both vertebral and nonvertebral fractures. Also, consistent with CHMP’s guidance on having a single Phase 3 study, the results from a single pivotal trial would need to be compelling. The pivotal study establishing the efficacy and safety of abaloparatide in postmenopausal women with osteoporosis was Study BA058-05-003 (ACTIVE) which was a randomized, double-blind, placebo-controlled, comparative Phase 3 multicenter study conducted in ambulatory postmenopausal women with osteoporosis and at risk for fracture [15]. The key efficacy endpoints in the ACTIVE study included the incidence of new vertebral and nonvertebral fractures with abaloparatide versus placebo following 18 months of treatment.

## Timelines for Regulatory Approval in the US and EU

**Figure 1.**
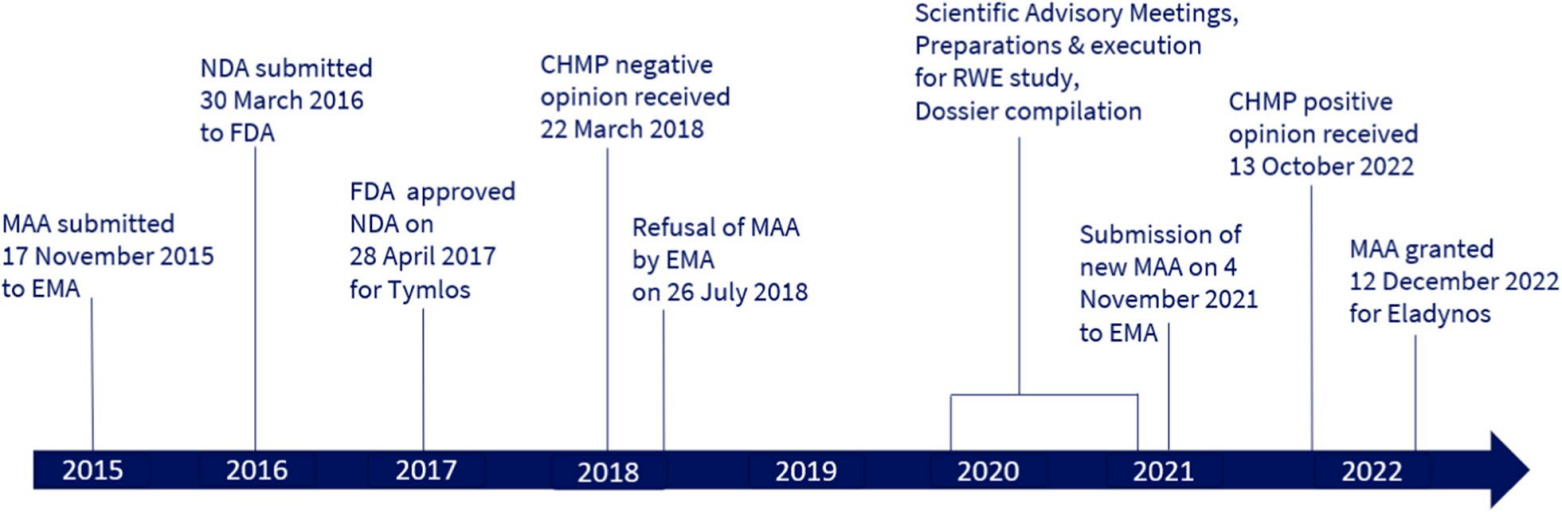
Timelines for Regulatory Approval in the US and EU.

The timelines for the submission and approval of the marketing authorization applications for abaloparatide in the US and EU are presented in Fig. 1.

Abaloparatide was approved in the US on 28 April 2017 under the tradename of Tymlos and is indicated for the treatment of postmenopausal women with osteoporosis who are at high risk for fracture (defined as a history of osteoporotic fracture or multiple risk factors for fracture), or patients who have failed or are intolerant to other available osteoporosis therapy. In postmenopausal women with osteoporosis, Tymlos reduces the risk of vertebral fractures and nonvertebral fractures. Abaloparatide was also recently approved by the FDA on 19 December 2022 as a treatment to increase bone density in men with osteoporosis at a high risk for fracture [17]. Abaloparatide was also approved in Japan on 23 March 2021 under the tradename of Ostabalo® for the treatment of male and female patients with osteoporosis who are at high risk for fracture.

On 17 November 2015, Radius International Ltd submitted an MAA for abaloparatide through the centralized procedure and received a negative opinion during the March 2018 CHMP meeting. A re-examination procedure started on 29 May 2018 and ended on 26 July 2018 with the refusal of the granting of the Marketing Authorization. The total review period of this dossier was 2 years and 7 months (from 4 December 2015 to 26 July 2018). There were two primary reasons for the overall negative benefit-risk assessment. First, only one pivotal study (BA058-05-003; ACTIVE) was conducted and due to serious Good Clinical Practice-related concerns associated with a single Principal Investigator, data from two sites were excluded, reducing the size of the study population by 16%. Consequently, the study failed to demonstrate a statistically significant effect on nonvertebral fractures (NVF) versus placebo. As shown in Table 2, abaloparatide did significantly reduce the frequency of NVF as compared to placebo following 18 months of treatment based on the full dataset. However, the sponsor agreed with the CHMP to exclude the data from these 2 clinical sites from the MAA.

**Table 2.** Nonvertebral Fracture Data from ACTIVE Presented with and without Data from the Two Excluded Sites.

	ACTIVE		ACTIVE, Excluding Two Sites	
	Abaloparatide (*n* = 824)	Placebo (*n* = 821)	Abaloparatide (*n* = 688)	Placebo (*n* = 696)
KM estimate event rate (%) (95% CI)	2.7 (1.7,4.3)	4.7 (3.4,6.6)	2.7 (1.6, 4.4)	3.6 (2.3, 5.4)
HR (95% CI)		0.57 (0.32, 1.00)		0.74 (0.38, 1.43)
*p* value		0.049		NS

*CI* confidence interval, *HR* hazard ratio, *KM* Kaplan–Meier, *NS* not significant

The totality of evidence included in the MAA supported that abaloparatide is effective in preventing nonvertebral fractures. Abaloparatide demonstrated trends toward the reduction of nonvertebral fractures (26%) and major nonvertebral fractures (46%), and abaloparatide significantly reduced major osteoporotic fractures by 69% (*p* = 0.004) versus placebo. An extension study was performed in which abaloparatide- or placebo-treated subjects from the ACTIVE study were treated with alendronate for an additional 2 years (Study BA058-05-005; ACTIVExtend) [18]. At 25 months, following 6 months of treatment with alendronate, subjects previously treated with abaloparatide (abaloparatide/alendronate) demonstrated a trend toward reducing nonvertebral fracture (48%), a significant 58% reduction in major nonvertebral fractures (*p* = 0.031), and a significant 63% reduction in major osteoporotic fractures (*p* = 0.017) versus placebo-treated subjects (placebo/alendronate). At 43 months (18 months of treatment with alendronate), abaloparatide/alendronate demonstrated a trend toward reduced nonvertebral fracture (39%), significantly reduced major nonvertebral fractures by 54% (*p* = 0.014), and significantly reduced major osteoporotic fractures by 52% (*p* = 0.024) versus placebo/alendronate. However, the failure to demonstrate a statistically significant effect on NVF versus placebo remained a major objection. The second major objection raised by the CHMP was related to concerns about potential safety risks associated with transient and reversible heart rate increases with abaloparatide compared to teriparatide and placebo. Because the pivotal study included relatively healthy ambulatory postmenopausal women free from significant cardiac disturbances, the data from the study were considered insufficient for assessing a risk for adverse cardiovascular outcomes potentially associated with transient increase heart rate in a generally more vulnerable real-world osteoporosis population of patients.

**Figure 2.**
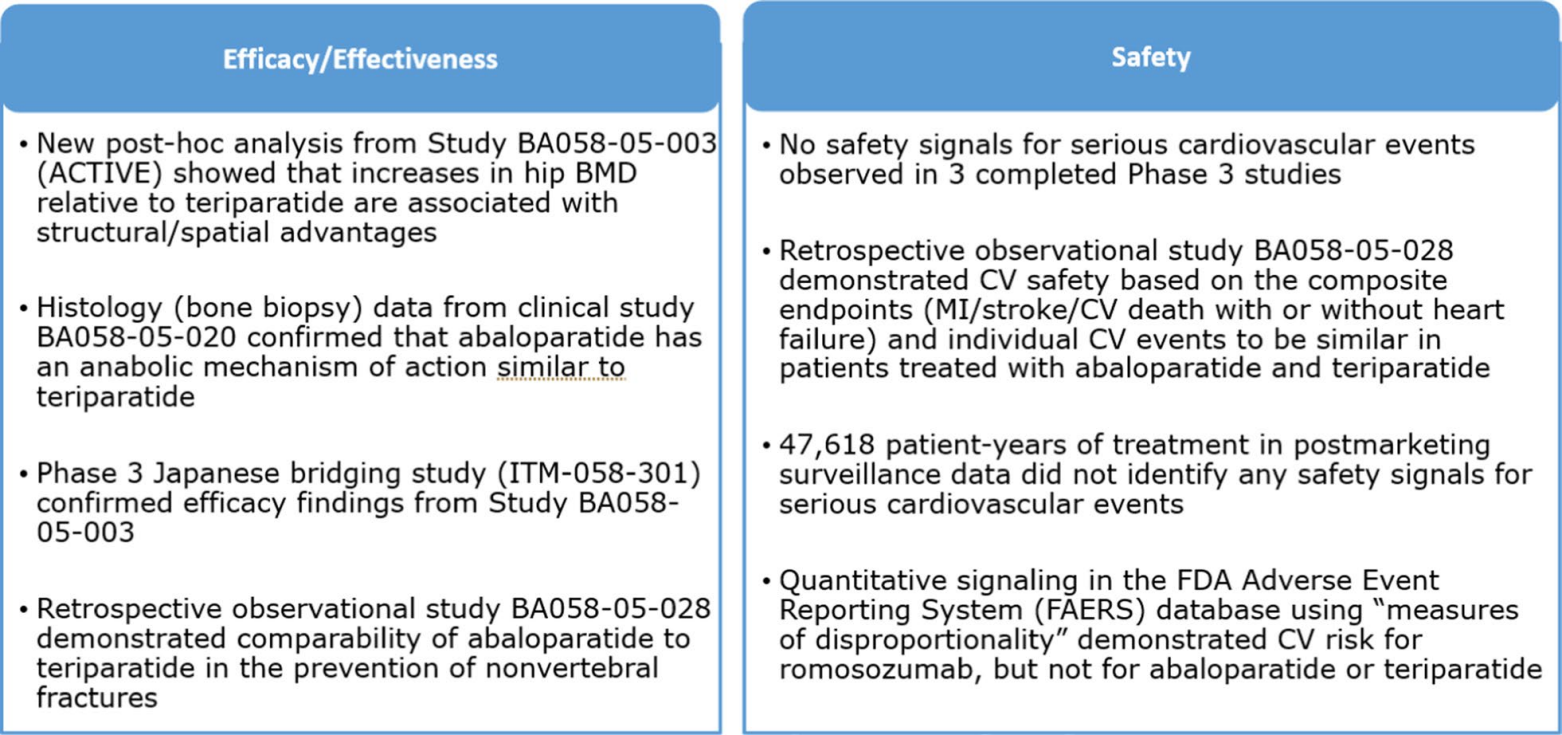
New Efficacy, Effectiveness, and Safety Data Address Concerns Raised from Prior EU MAA Review.

Since this initial application, new clinical data have been acquired via the conduct of additional interventional and observational studies, as well as post-marketing data obtained from the US market since approval of abaloparatide on 28 April 2017 (see Fig. 2).

These data included (1) a dual-energy X-ray absorptiometry (DXA)-3D-based modeling study (post-hoc analysis in a sub-population of the ACTIVE study [19]) to further differentiate the effects of abaloparatide on hip cortical volumetric BMD and estimated hip strength; (2) a histomorphometry study in women with osteoporosis (BA058-05-020) that further demonstrated the mechanistic action and anabolic effect; (3) a Japanese Phase 3 bridging study providing additional safety and efficacy data and further understanding of the effects on hip biomechanical properties (ITM-058-301); (4) a retrospective observational cohort study (BA058-05-028) using data from the Health Claims database (Symphony Health Integrated Dataverse (IDV)®) with a pre-specified protocol and statistical analysis plan demonstrated that abaloparatide was comparable to teriparatide for prevention of NVF, resulted in a 22% risk reduction for hip fractures, and demonstrated similar cardiovascular safety following 18 months of treatment [20]; and (5) post-marketing surveillance information from the US demonstrating no cardiovascular safety signal. These data enhanced the understanding of abaloparatide’s mode of action in humans and provided additional evidence on the safety and efficacy of abaloparatide to reduce fracture risk at both vertebral and nonvertebral sites.

Prior to the planned resubmission in EU, Radius obtained national SA from countries that previously had both positive and negative assessments for the initial MAA including MPA/Sweden (28 January 2021), AGES/Austria and BfArM/Germany (17 February 2021), Lithuania (17 March 2021), NoMA/Norway (18 March 2021), SNS/Portugal (8 April 2021), FAMHP/Belgium (13 April 2021), and AEMPS/Spain (26 April 2021). The goal for the SA was to obtain feedback on the proposed overall clinical program to be used for the new MAA submission, specifically on the relevance of real-world data collected in a proposed retrospective study to support the efficacy and safety assessment of prospective clinical trials. A summary of the key points raised during these meetings is summarized in Table 3.

**Table 3.** Summary of Scientific Advice.

Topic	Key Points Raised During Meeting
Efficacy	Justify why it is not possible to conduct a second RCT to collect fracture data
	The observational cohort study is not powered to show equivalence between abaloparatide and teriparatide but presents a lack of difference. Therefore, must demonstrate external validity of results and that abaloparatide is clearly distinguished from placebo with a robust effect on NVF
	Address the clinical relevance rather than the lack of statistical significance with respect to NVF
	Describe the clinical relevance of the change in bone mineral density from the 3D-DXA study in the dossier
	Show the correlation between BMD, bone strength, and fracture risk reduction in the dossier
	Anabolic properties and BMD data are reassuring and corroborate clinical efficacy. Literature data provides very strong evidence that BMD can be a surrogate endpoint and may even be used in future clinical trials as an endpoint [21]
Safety	RWE should include subgroup analysis for patients with high cardiovascular (CV) risk and very high CV risk
	Explain the mechanism for tachycardia and hypotension in the dossier
	Provide as much information as possible on the 70 cases of death noted in the post-marketing safety data for which the cause of death was unknown
	Provide data in the dossier on impact of abaloparatide on arrhythmia
Observational RWE Study	Clearly define all methodology including pre-specification of criteria for analysis, covariates, analysis methods, and sensitivity analyses
	Include a predefined statistical plan, protocol, and all planning documents
	Clearly define variables used for propensity score matching to make sure that the populations are comparable
	Discuss the strengths and limitations of the study
	Discuss potential for bias and methodological approach to minimize biases in the study
	Describe why US patients are relevant for the EU population
	Recommend collecting mortality data
	RCT is still the gold standard and RWD may not be able to support approval of a new product; RWE can be used as supportive information
	The applicability of the RWD will depend on the quality of the data

Most agencies were supportive of a new MAA submission and considered RWE an important part of the dossier. There was no doubt that abaloparatide had an anabolic effect on bone, but the question remained whether this would translate into a clinical effect on reduction of NVF. The SA was divergent on whether data from an observational cohort study could provide adequate evidence that abaloparatide decreased the risk of NVF; and in the absence of conducting a second RCT, it was clear that this would be a critical review issue.

The sponsor included a justification in the MAA for why a second RCT was not performed. This justification was based on the totality of information collected with abaloparatide (pivotal and supportive studies; see Fig. 2) as well as the knowledge of the effects of teriparatide (same molecule class, same mode of action). The pivotal ACTIVE study demonstrated that treatment with abaloparatide resulted in a significant reduction in VF versus placebo and a consistent trend in favor of reduction of NVF. There was no scientific reason to presume efficacy only for VF and not on NVF, especially considering the relevant increases in BMD observed at the lumbar spine, hip, and femoral neck. Therefore, considering all the available data, the sponsor considered it inappropriate to conduct a second placebo-controlled study in subjects at high risk for fracture but worked with the health authorities to prospectively design an observational study to support the comparable effectiveness of abaloparatide versus teriparatide of NVF rate in high-risk patients, and to corroborate the findings of the pivotal ACTIVE study.

Regarding safety, several of the agencies indicated that it would be important to include data on mortality as part of the observational study. It was also highlighted that it was important to present not only the major adverse cardiovascular event (MACE) data but also data on tachycardia and arrhythmia. Because the main safety concern with abaloparatide would be for patients with high or very high CV risk, it was recommended to look at these subpopulations. Regarding the observational study, advice also stressed the importance of providing all protocols and planning documents to avoid any concern that the research approach was data driven. The dossier should include all the information available to support that the analyses had been pre-specified.

Sensitivity analyses were requested to demonstrate the stability of the propensity score-matched cohorts, and to evaluate the impact of prior non-anabolic treatments and duration of anabolic treatment on the effectiveness and safety endpoints. For cardiovascular safety evaluation, sensitivity analyses were also requested to test the robustness of findings by baseline CV risk factors given the high prevalence of these risk factors in the target population. During the review of the MAA, the CHMP requested additional sensitivity analyses including the original data without propensity score matching, propensity score stratification, and inverse probability of treatment using the propensity score and multivariable regression model to the original data adjusting for all confounders. The CHMP also requested Intention-To-Treat (ITT) analysis for safety endpoints, and an As-Treated (AT) analysis for effectiveness evaluation.

A discussion of the strengths and limitations of RWE was also requested during scientific advice.

## Study BA058-05-028: A Retrospective, Observational Cohort Study Evaluating the Effectiveness of Cardiovascular Safety of Abaloparatide in Postmenopausal Women New to Anabolic Therapies

A retrospective observational cohort study was conducted using anonymized patient claims data from PRA Health Science’s Symphony Health Integrated Dataverse (IDV) database including the enhanced hospital data (NCT04974723). The goal of this observational study was to evaluate the comparative effectiveness and cardiovascular safety of abaloparatide versus teriparatide for the treatment of osteoporosis in postmenopausal women in the real-world healthcare setting in the US. The methodology and results from this study have been previously published and are briefly summarized below [20].

Data for this study included routinely collected information in healthcare encounters from all available healthcare settings (inpatient hospital, outpatient hospital, physician office, pharmacy, etc.) for all types of provided services including specialty, preventive care, and office-based treatments. Since this was not a randomized study, patients were matched using an extensive list of indicators of disease severity and fracture risk, including fracture and treatment history as per evidence-based clinical practice guidelines [22] by logistic regression-based propensity score (PS) matching in order to ensure that the two cohorts were comparable in their probability of receiving and benefiting from treatments. A total of 11,027 patients were included in both the abaloparatide and teriparatide groups. The post-index treatment period consisted of the 18 months after the index date (date initial prescription dispensed) with the maximum evaluation period of 18 months plus 30 days of follow-up (19 months), to be consistent with the pivotal Phase 3 study (ACTIVE).

The primary analyses of effectiveness were based on the time to first incidence of NVF. From a clinical perspective, any reduction in risk of NVF versus placebo that is greater than 25% is clinically important since this would indicate an improvement in the effect for bisphosphonates, as seen in the HORIZON trial [23]. Overall, 313 (2.8%) patients in the abaloparatide cohort and 333 (3.0%) patients in the teriparatide cohort had a NVF. The risk of new NVF from the index date was similar between the abaloparatide and teriparatide groups [HR (95% CI) 0.94 (0.81, 1.10)]. With regard to hip fractures, 112 (1.0%) and 125 (1.1%) patients in the abaloparatide and teriparatide cohorts, respectively, had a hip fracture. The risk of new hip fractures from the index date was similar between the two cohorts [HR (95% CI) 0.90 (0.69, 1.16)].

Cardiovascular safety was evaluated by examining the time to first incidence of a composite endpoint of MI, stroke, and hospital CV death, with and without inclusion of heart failure. The incidence of the composite endpoint of MI, stroke, and hospital CV death was similar in abaloparatide and teriparatide-treated cohorts (2.0% and 1.9% with abaloparatide and teriparatide, respectively, [HR (95% CI) 1.08 (0.89, 1.30)], as was the incidence of the composite endpoint of MI, stroke, heart failure, and CV death (4.5% and 4.3% with abaloparatide and teriparatide, respectively, [HR (95% CI) 1.08 (0.95, 1.22)]). The individual events of MI, stroke, heart failure, hospital CV death, and hospital all-cause death also occurred with similar frequency between the abaloparatide and teriparatide-treated cohorts.

The observational RWE study had several strengths allowing for a robust comparative assessment of treatment effectiveness and safety. The data are representative of a broad population of patients including those with more CV risk factors compared to the subjects included in the randomized controlled pivotal Study BA058-05-003. Because there are no restrictions for CV disease in the US labeling for abaloparatide (Tymlos), approximately 76% of patients in the BA05-05-028 study had CV risk factors and approximately 10% of patients had a prior event of MI, stroke, or heart failure. In addition, data were from multiple payers and geographically diverse settings across the US and captured over 90% of pharmacy claims in the US. The prescription claims were for prescriptions filled, not prescriptions written. As such, the findings from the study were expected to have a high generalizability. A claims-based fracture incidence algorithm, which has a high specificity and has been shown to have over 90% accuracy based on positive predictive value in previous studies [24], was used to assess fracture events. In addition, because over 11,000 patients were included in each cohort, a much larger number of NVF were observed in the BA058-05-028 study as compared to the pivotal BA058-05-003 study.

## CHMP Review of 2021 MAA

Following receipt of the D120 List of Questions (LoQ), it was clear that the original evidence gap regarding effect of abaloparatide on NVF had been addressed. While it was pointed out that the effect of abaloparatide versus placebo on NVF was not statistically significant in the pivotal Phase 3 ACTIVE study, the data were indicative of a trend in favor of abaloparatide. Regarding the fact that a second pivotal study was not conducted, the CHMP reviewers considered that the additional data submitted including a placebo-controlled Phase 3 bridging study in Japan, a histomorphometry study in patients confirming the anabolic mechanism of action, and new post-hoc analyses of the hip DXA images from the ACTIVE study providing data on hip cortical volumetric BMD and estimated hip strength to be supportive of efficacy. The CHMP also considered the NVF data from the > 22,000 patients included in the RWE observational cohort study including more than 646 NVF to be supportive. The CHMP raised several concerns related to the conduct and analysis of this observational study and requested several sensitivity analyses to be conducted. The CHMP stated in the EPAR that while “there were important limitations in the design of the observational study, the main analyses as well as several sensitivity analyses support comparable effectiveness versus teriparatide in a US population to a degree that indicates superiority to a putative placebo.”

Regarding safety, the Day 120 LoQ included one major objection regarding the potential risk of serious cardiovascular events due to the transient increase in heart rate with abaloparatide compared to placebo. The CHMP acknowledged that this MAA included post-marketing data from the US covering 47,618 patient-years of treatment and that no signal for MACE had been observed. Due to the outstanding questions related to the conduct and analysis of the observational study, the reviewers could not conclude if these data would address the remaining issue of cardiovascular safety.

The sponsor submitted a response to the Day 120 LoQ including safety data from a recently completed Phase 3 study in men with osteoporosis (BA058-05-019) as well as the requested sensitivity analyses for the observational RWE study. In the Day 180 List of Outstanding Issues (LoOI), the Major Objection had been resolved. In the EPAR, the CHMP indicated that the Japanese bridging study and new histomorphometry study did not provide any additional insights in the cardiovascular safety of abaloparatide. Data from Study BA058-05-019 suggested that men and women treated with abaloparatide had a similar frequency and pattern of CV events with no new safety concerns being identified. Regarding the observational study (BA058-05-028), the CHMP stated that “that the MACE (MI/Stroke/heart failure/hospital CV death) event rates were not significantly increased in abaloparatide treated patient compared to teriparatide.” The CHMP cited the main study limitations to be the absence of mortality data recorded outside of hospitals, lack of access to income and education data, and potential bias caused by difference in price between abaloparatide and teriparatide (abaloparatide is less expensive). In the EPAR the CHMP stated, “the new data has not confirmed that increases in heart rate associated with abaloparatide would be associated with increase of serious CV events. However, both the observational study and post-marketing data have important limitations. Still, for a majority of osteoporosis patient, abaloparatide is well tolerated and the safety seems acceptable.”

The ongoing concern that patients with untreated heart disease or rhythm disturbances may still be at risk of serious adverse events was to be addressed in the labeling and the eventually agreed upon Post Authorization Safety Study (PASS). On 12 December 2022, the EU granted marketing authorization of abaloparatide under the tradename of Eladynos® to treat osteoporosis in postmenopausal women at increased risk of fracture. On 27 March 2023, the Medicines & Healthcare products Regulatory Agency (MHRA) approved Eladynos® for the same indication in the Great Britain under the European Commission Decision Reliance Procedure.

## Discussion

This case study is one of the first examples of RWE used to support the approval of a new medicinal product in the EU for a disease state not considered to have high unmet medical need and a non-orphan indication. The CHMP assessed data from a single pivotal Phase 3 study, clinical data from additional interventional and observational studies, as well as post-marketing data obtained from the US since the approval of abaloparatide in 2017. The new interventional studies were not designed to assess fracture efficacy and cardiovascular safety which were topics of concern raised by the Committee for Medicinal Products for Human Use (CHMP) during their review of the initial MAA submitted in 2015. However, data from the observational study addressed the previous CHMP concern regarding the effectiveness of abaloparatide to treat osteoporosis in postmenopausal women with an acceptable cardiovascular risk profile. This study was designed in accordance with available FDA and EMA guidance and SA from eight national Health Authorities in which the decision makers’ perspective on the robustness of the source of data, methodology, and addressing biases associated with real-world study design were considered.

While RWE studies are not meant to replace RCT, fit-for-purpose data can be used as supporting evidence when conduct of an additional RCT is not feasible or is unethical. Due to the totality of the available efficacy data for abaloparatide, the sponsor considered conducting a second placebo-controlled study to generate additional efficacy data based on a reduction in the incidence of NVF to be impractical and possibly unethical in subjects at high risk for fracture. An important advantage of observational studies is the inclusion of a larger population of patients than normally seen in a RCT and a larger number of events of interest. Indeed, the observational study that demonstrated comparable effectiveness between abaloparatide and teriparatide included more than 22,000 patients with almost 650 NVF. Abaloparatide and teriparatide are both anabolic drugs that stimulate new bone formation following binding to the PTH1 receptor. As stated in the EPAR, the CHMP considered that the data from the observational study to support comparable effectiveness versus teriparatide to a degree that indicated superiority to a putative placebo. An additional advantage of the observational study is that the evaluation of safety outcomes is not restricted to a small population of patients with few comorbidities as in the RCT. The CHMP had considered the pivotal Phase 3 ACTIVE study insufficient for assessing CV risk potentially associated with transient increase in heart rate because subjects were primarily healthy ambulatory postmenopausal women free from significant cardiac disturbances. In the EPAR, the CHMP indicated that while the new post-marketing and observational data did not confirm that increases in heart rate were associated with an increase of serious CV events, the safety seemed to be acceptable for a majority of osteoporosis patients.

It is unusual for RWE to support approval of a new indication since real-world data are not available until after regulatory approval and market access. However, in the current case study, abaloparatide was approved previously in the US, where more than 5 years of real-world data had been accumulated and were available for research. Furthermore, an argument was made for the generalizability of the findings from the US to the EU population based on comparative epidemiology, treatment patterns, and global clinical practice guidelines [22, 25, 26]. In addition, data from the pivotal Phase 3 ACTIVE study showed that pharmacokinetics and pharmacodynamics were not different between EU and US regions, and intrinsic factors such as age, race, region, weight, prior fracture, and severity of disease did not affect efficacy or safety of abaloparatide with similar results seen across the four geographies including the US and EU.

Despite the above argument, the CHMP still considered there to be a limitation of using data from one country for another due to differences in health care delivery and potential differences in the abaloparatide target population characteristics between US and EU populations (e.g., age, severity of disease). A post-marketing study using European registries to assess CV risk was considered to be feasible as such studies had previously been performed for other osteoporosis medicines. The CHMP believed that such a Post-approval Safety Study (PASS) may provide a more comprehensive safety dataset including all-cause mortality data. Therefore, a PASS was proposed as part of the Risk Management Plan for Eladynos in the EU.

Since the early use of RWE in the post-approval safety space, RWE has more recently been accepted as supporting evidence in the benefit–risk evaluation of new indications or even initial approval of new medicinal products by global health authorities. The usefulness of RWD for regulatory decision making is dependent on data quality, pre-specification, and transparency of methodology, consideration of methodological approaches to minimize bias, and early discussions with health authorities to ensure that the data are fit for purpose. Flynn et al. [9] and Bakker et al. [10] described use of RWE in regulatory decision making for new MAAs submitted via the centralized procedure between 1 January 2018 and 31 December 2019. Most of these applications were for indications of high unmet need or orphan designation. In a recently issued report from the EMA [8] on regulator-led real-world studies, safety evaluation was the most cited utility of such data for the regulatory review. The case study presented here is unique because the RWE was used to support the initial approval of a new medicinal product for osteoporosis in the EU. Another recent regulatory case study [27] described a retrospective cohort study used to demonstrate that the effectiveness and safety of denosomab (tradename Prolia) in Chinese women living in Taiwan and Hong Kong was comparable to the data from the global Phase 3 pivotal fracture study (FREEDOM) leading to the initial approval of denosomab in China.

In conclusion, the EMA is increasingly considering RWE in their review of MAAs and in the case of abaloparatide, the EMA recently considered the totality of evidence in a newly submitted MAA, which included data from a prospectively planned retrospective observational cohort study, leading to the approval of a new medicinal product for osteoporosis. This regulatory case study demonstrated that with early scientific advice, it is possible to use real-world data to address previously raised concerns regarding benefit–risk leading to approval of a new medicinal product for an indication not associated with an orphan designation or high unmet need in the EU.

## Data Availability

The source data for this study were licensed from a third party, by Radius Health, Inc (Radius). Although we are not permitted to share the licensed data publicly, the same data used in this study are available for others to license by contracting with the database owners. Radius licensed data from Symphony Health, Integrated Dataverse (IDV)®, May 2012 to January 2021, which included anonymized patient level data from pharmacy claims linked to commercial and Medicare medical claims data. Symphony Health licensing information can be found at www.symphonyhealth.com with the database owners. The authors did not have any special access privileges that other parties who license the data and contract with Symphony would not have.
